# Comparison of Patient-Specific Condylar Positioning Devices and Manual Methods in Orthognathic Surgery: A Prospective Randomized Trial

**DOI:** 10.3390/jcm13030737

**Published:** 2024-01-27

**Authors:** Anton Straub, Sebastian Gubik, Alexander Kübler, Niko Breitenbuecher, Andreas Vollmer, Tobias Renner, Urs Müller-Richter, Stefan Hartmann, Roman Brands

**Affiliations:** Department of Oral and Maxillofacial Plastic Surgery, University Hospital Würzburg, Pleicherwall 2, 97070 Würzburg, Germany

**Keywords:** ascending ramus, bilateral sagittal split osteotomy, CAD/CAM, condylar positioning device, temporomandibular joint, virtually planned position

## Abstract

Background: This study investigated whether patient-specific condylar positioning devices (CPDs) are beneficial compared to the conventional manual positioning of the condyles. Methods: In this prospective, randomized trial, patients undergoing orthognathic surgery with a bilateral sagittal split osteotomy of the mandible were included. The ascending ramus was positioned with computer-aided designed and computer-aided manufactured (CAD/CAM) patient-specific devices in the CPD group and manually in the control group. Postoperatively, cone-beam computed tomography (CBCT) was performed to align the virtually planned position with the postoperative result. Results: Thirty patients were enrolled in the study, with 14 randomized to the CPD group and 16 to the control group. In the CPD group, the ascending ramus differed in the postoperative CBCT scan from the virtually planned position by 0.8 mm in the left/right, 0.8 mm in the front/back, and 1.3 mm in the cranial/caudal direction. The corresponding control-group values were 1.1 mm, 1.3 mm, and 1.6 mm. CPD and controls differed significantly for the left/right movement of the rami (*p* = 0.04) but not for the other directions or rotations (*p* > 0.05). Conclusions: The results demonstrate that both methods are accurate, and postoperative results matched the virtually planned position precisely. It can be assumed that the described CPDs are beneficial when a condylar position different from the preoperative is desired.

## 1. Introduction

The condylar position of the ascending ramus continues to be the subject of discussion in orthognathic surgery (OS). Several studies have described changes in the condylar position after OS [1,2,3]. Most studies conclude that the changes are small (approx. <2 mm, <5°) and the clinical relevance is low. The effect on the development of temporomandibular joint disorders (TMD) is also seen as a controversial issue in discussion. While some authors do not see a correlation between condylar position and the development or aggravation of TMD symptoms, others demonstrate exactly this [4,5,6,7,8]. It is known that the malpositioning of the condyle can cause TMD symptoms, clicking, and crepitation in the joint [5]. Several condylar positioning devices (CPDs) have been developed in the past. For example, Landes et al. revealed in a prospective trial that the use of CPDs significantly reduces postoperative dysfunction, disc dislocations, and skeletal relapse [9]. Furthermore, the right condylar position is crucial to ensure the stability of the surgical procedure and to prevent early and late relapse [2,10]. Early relapse is mainly caused by the incorrect placement of the condyles in their fossa. If the condyle, for example, is placed too ventrally, the mandible will deviate when the patient is awake, and the condyles will find their way back to their centric position. This particular event is actually not a true relapse but a primary incorrect osteosynthesis of the mandible. The placement of the condyles too dorsally in the fossa can cause remodelling and resorption, which results in a late relapse. Thus, the correct positioning of the condyles ensures the stability of the surgical result [2,5,11]. Taking this into account leads to the assumption that CPDs should be used in OS, because they can counteract such malpositioning. However, the most commonly performed technique is the manual positioning of the condyles in their fossa without the help of any devices such as CPDs [12]. The main reasons for this are that the use of CPDs is time-consuming and cumbersome and that studies have mainly failed to demonstrate any beneficial effect [11,12,13].

Furthermore, the preoperative condylar position is not necessarily the desired postoperative position [12]. To harmonize the mandibular basal margin, it may be useful in some cases to rotate the ramus anteriorly (pitch). In other cases, mandibular advancement or setback can require the lateral rotation of the ascending ramus (roll and yaw). Furthermore, in patients with symptoms of TMD or a diagnosis of arthrosis, a different condylar position may be desirable [12]. In the past, CPDs were mainly used to conserve the preoperative position, and it was not possible to transfer a preoperatively newly planned condylar position to the operating room [6].

In the last few years, computer-aided designed and computer-aided manufactured (CAD/CAM) cutting guides have helped in solving the above-mentioned problem and enabling the smooth transfer of a position planned prior to surgery using a virtual-reality-based software environment (the virtually planned position) directly to the operating room. CAD/CAM-fabricated CPDs have also been described, but these devices still focus on preserving the preoperative condylar position rather than a patient-specific autorotated and planned position [14,15,16,17,18]. In current times, in which OS is planned virtually and a physiological condylar position can be designed in the software prior to surgery, a CPD that transmits the virtually planned position to the operating room can be very helpful. We thus developed patient-specific CPDs by modifying the final occlusal splint, which should allow for the exact positioning of the ascending ramus according to the planned position without complicating and prolonging the operation itself. Especially for trainees and less experienced surgeons, in difficult cases with a major displacement of the mandible, in patients with facial asymmetries, or in patients without a clear centric condyle position (Sunday bite), these CPDs could ensure the stability of the surgical result and prevent relapses. In addition, a condylar position different from the preoperative situation is perhaps desirable in patients with TMD symptoms or temporomandibular joint arthritis. Hence, the aim of the present study was to investigate whether patient-specific CPDs that allow for the transfer of a virtually planned condylar position prior to surgery to the operating room are indeed beneficial compared to the conventional method of manual positioning.

## 2. Materials and Methods

### 2.1. Study Design

This prospective, two-arm, and open-label single-centre study included patients who underwent orthognathic surgery, and more specifically a bilateral sagittal split osteotomy (BSSO) of the lower jaw. The study was conducted at the Department of Oral and Maxillofacial Plastic Surgery at the University Hospital in Würzburg between May 2021 and March 2022. Inclusion criteria were:

- Age of 18 or older
- Patients undergoing orthognathic surgery in the form of BSSO of the mandible either as part of a bimaxillary operation or an isolated osteotomy of the mandible
  Exclusion criteria were:
- Patients who failed or were unable to comply with the study protocols after inclusion (e.g., intraoperative complications like bad split, operation splint failure, or an evaluation of cone-beam computed tomography (CBCT) scan proving impossible)

**Figure 1:**
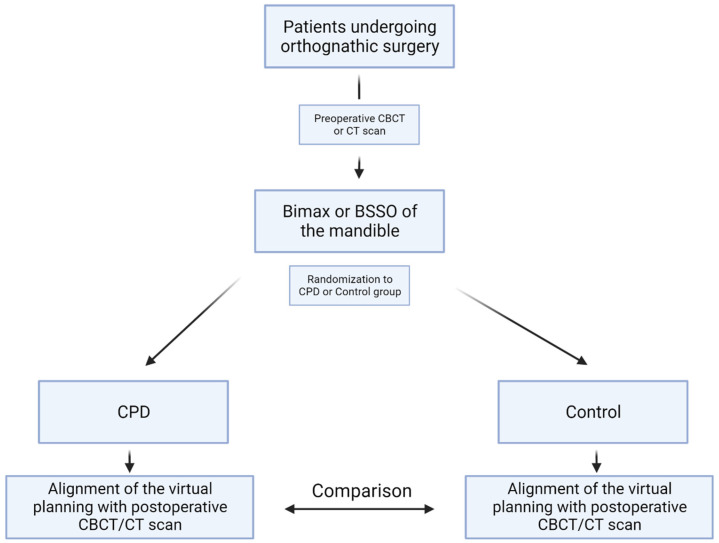
Flowchart of the study protocol. Patients undergoing orthognathic surgery in the form of a BSSO of the mandible were included and randomized either to the CPD or to the control group. Postoperative CBCT scan and the virtually planned position were aligned and differences measured. Abbreviations: BSSO: bilateral sagittal split osteotomy, CPD: condylar positioning device, CBCT: cone-beam computed tomography, CT: computed tomography.

Patients were randomized into the intervention (CPD) or the control group using a simple randomization sheet. Patients in both groups underwent surgery with the same technique and manner (see Section 2.2). In the intervention group, patient-specific CPDs were used additionally (Figure 1).

The protocols implemented in this study were approved by the institutional review board of the University of Würzburg (IRB approval numbers “223/20-am”). The study was conducted in accordance with the Declaration of Helsinki, and all participating patients provided written informed consent.

### 2.2. Planning and Surgical Technique

Patients undergoing orthognathic surgery received a preoperative CBCT scan, and a dental impression of the maxilla and mandible was taken. The preoperative CBCT scan was performed with a wax bite in the centric condyle position [19]. All operations were planned virtually with IPS CaseDesigner (KLS Martin Group, Tuttlingen, Germany). The ascending rami of the mandible were autorotated (pitched) to harmonize the mandibular basal margin. Furthermore, (pronounced) mandible setback and advancement needed an adjustment of the ramus position (yaw or roll) in some cases. Planning was completed by generating the occlusal splints.

BSSO was performed according to the modified technique described by Obwegeser and Hunsuck/Epker in all patients [20]. In the case of a bimaxillary procedure, a LeFort I osteotomy of the upper jaw was performed first. Occlusal splints (final splint and, in cases of bimaxillary operation, intermediate and final splint) and intermaxillary wiring were used to obtain the correct occlusion. For intermaxillary wiring orthodontic applications or osteosynthesis screws were used. A rigid osteosynthesis of the upper jaw was performed with four mini plates (Midface plate Modus 2 M2-7042, 0.6 mm, Medartis, Umkirch, Germany) and of the lower jaw with a conventional mini plate (Sagittal Split Plate Modus 2 M2-4050, 0.8 mm, Medartis, Umkirch, Germany) on each side. In the intervention group, the patient-specific CPDs were used instead of the conventional final splint to position the ramus and during osteosynthesis. Correct occlusion was verified with and without the conventional final splint in both groups. All splints used were printed with a Form 3+ printer (Formlabs GmbH, Berlin, Germany) and surgical guide resin (Formlabs GmbH, Berlin, Germany).

### 2.3. Manual Condylar Positioning

Condyles in the control group were manually positioned. By forcing the condyle to a superior position and releasing the force, a reproducible and steady position in the center of the glenoid fossa was found [5]. The ascending ramus was manually rotated (pitched, yawed, and rolled) according to the virtually planned position.

### 2.4. Condylar Positioning Devices

**Figure 2:**
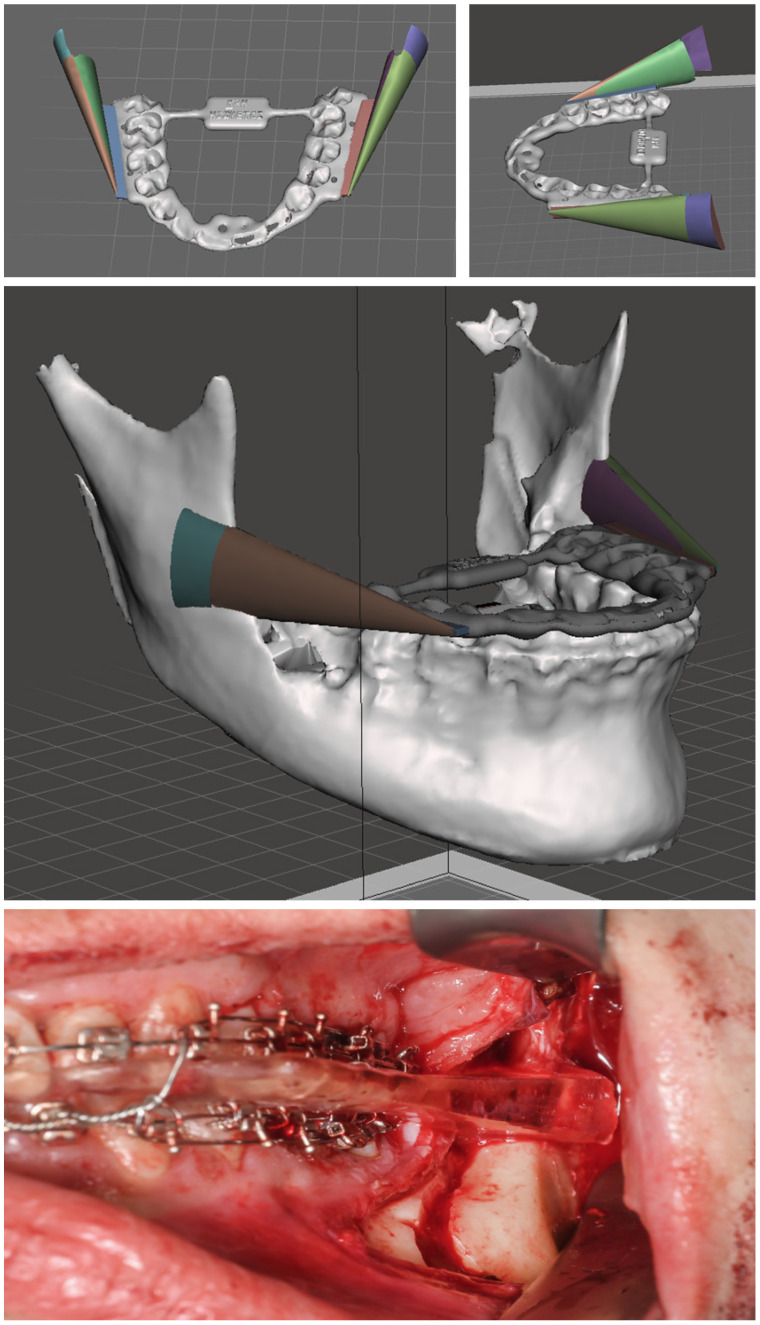
Patient-specific CPD to transmit the position of the ascending ramus planned virtually to the intraoperative site. In this patient, the mandible was set back 1.2 mm. The two wings, which reflect on their inner side the new position of the ramus, were attached to the final occlusal splint. The picture at the bottom illustrates the intraoperative site with the inserted CPD and intermaxillary wiring.

The patient-specific CPD was designed with the open-source software Meshmixer (Autodesk, San Francisco, CA, USA). The operation was planned virtually with IPS CaseDesigner (KLS Martin Group, Tuttlingen, Germany), in which the STL files of the operated jaw were generated and subsequently exported. The final splint and the operated mandible were imported to Meshmixer (Autodesk, San Francisco, CA, USA). With preformed tools, an additional wing was attached to each side of the final splint. The surface of the ascending ramus was imprinted using a Boolean function. The modified splint thus reflected the planned position of the ramus (Figure 2).

### 2.5. Intra- and Postoperative Management

Patients were treated as inpatients at University Hospital Würzburg. They received a single shot of cefazolin 2 g (Hikma, London, UK) and metronidazole 500 mg (B. Braun Supply Solutions, Melsungen, Germany), or ampicillin/sulbactam 3 g (Unacid^®^, Pfizer Pharma GmbH, Berlin, Germany), or clindamycin 600 mg (Sobelin^®^ Solubile, Pfizer Pharma GmbH, Berlin, Germany) intraoperatively, as well as up to 48 h following surgery (cefazolin, ampicillin/sulbactam, and clindamycin administered three times daily, and metronidazole once daily in case of administration in the postoperative course).

The CBCT scan was performed according to a standardized protocol a few days after the operation during the hospital stay depending on the degree of swelling, with the final splint and intermaxillary fixation with elastic bands inserted.

Patients maintained a soft diet for six weeks. The elastic bands remained in place for two to three weeks. We recommended each patient to have the metal plates removed after six to nine months.

### 2.6. Measurement

**Figure 3:**
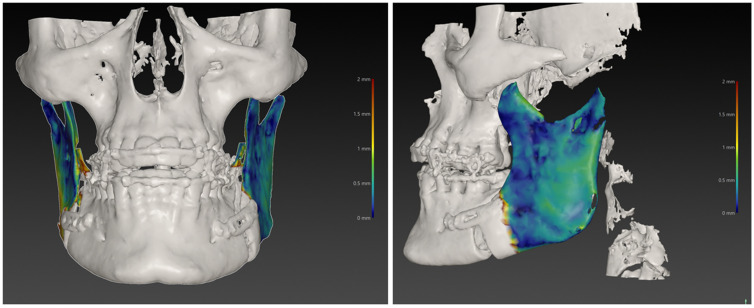
Heatmap depicting differences between the virtually planned position and the postoperative CBCT scan in a patient with mandibular setback. Blue corresponds to regions of exact and red to regions of poor matching.

Via IPS CaseDesigner (KLS Martin Group, Tuttlingen, Germany), the position of the left and right ramus planned virtually was compared with the postoperative outcome. A heat map was generated (Figure 3) depicting differences between the planned position and postoperative results in millimetres and degrees.

### 2.7. Data Processing and Statistics

To calculate sample size, a power analysis was performed a priori using the G*Power software (Version 3.1, Düsseldorf, Germany) with α = 0.05, β = 0.20, and a power of 0.8. All further statistical analyses (descriptive statistics, Mann-Whitney test, and Student’s *t*-test) were performed with GraphPad Prism (Version 10, San Diego, CA, USA). The Shapiro–Wilk test was used to test for normality. If the Shapiro–Wilk test proved to be significant (*p* < 0.05), a two-tailed Mann–Whitney test was performed.

## 3. Results

Thirty patients were eligible for inclusion, of whom 14 were allocated to the intervention and 16 to the control group. The mean age was 23 in the intervention group and 26 in the control group. The proportion of females was greater in both groups. Table 1 presents the descriptive statistics of the study population.

Patient characteristics (age, gender) and performed operation did not differ significantly between both groups (*p* > 0.5 with Student’s *t*-test and Mann–Whitney test). The displacement of the mandible, operation time, and number of necessary surgical revisions did not differ significantly in both groups (*p* = 0.2, *p* = 0.2, *p* = 0.2, Mann–Whitney test).

### 3.1. Ramus Position: Comparison of Postoperative Results with the Virtually Planned Position

**Figure 4:**
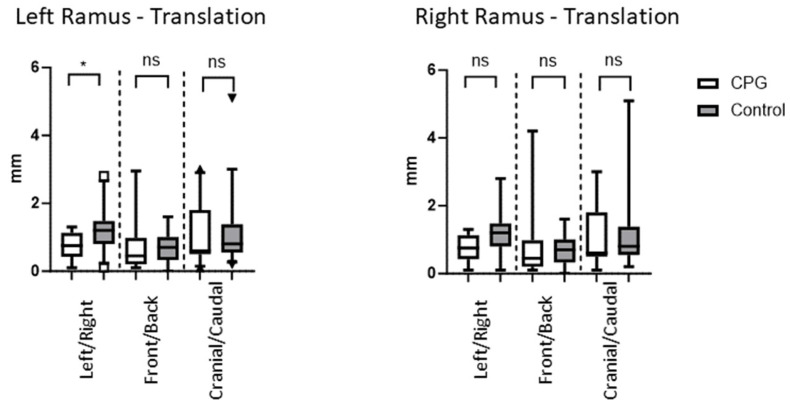
The deviation of the ascending ramus is given in millimetres. The CPD appears to be slightly more precise; however, differences were only significant for the left/right translation of the left ramus (*p* = 0.04 with Mann–Whitney test). * = significant result, ns = non-significant result.

The differences in the translation of the ramus between the virtually planned position and the postoperative results were 0.8 mm, 0.8 mm, and 1.3 mm (left/right, front/back, cranial/caudal, respectively) in the intervention group in which the CPD was employed. In the control group, these values were slightly higher at 1.1 mm, 1.3 mm, and 1.6 mm, respectively. Differences between the two groups, in favour of the CPD, were only significant for the left/right translation [*p* = 0.04 with unpaired and two-tailed *t*-test (Table 2 and Figure 4)].

**Figure 5:**
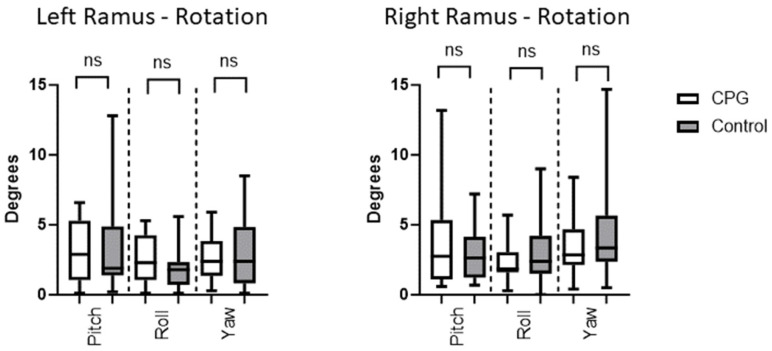
The rotation of the ramus is measured in degrees and depicted for the left and right ramus separately. Values in the CPD and control group were comparable without any significant difference between both groups. ns = non-significant result.

The differences in the rotation of the ramus between the virtually planned position and the postoperative results were 2.4°, 3.3°, and 3.0° (pitch, roll, and yaw, respectively) in the intervention group in which the CPD was used. In the control group, these values were 2.4°, 3.1°, and 3.7°, respectively. Differences between both groups proved to be statistically insignificant [*p* > 0.05 in both unpaired and two-tailed *t*-test (Table 3 and Figure 5)].

### 3.2. Comparison of Translations and Rotations in Mandibular Advancement and Setback

Differences between patients with mandibular advancement and setback for left/right, front/back, and cranial/caudal translations were not significant (*p* = 0.3, *p* = 0.7, *p* = 0.1, Mann–Whitney test). Furthermore, we found no difference in yaw and roll between mandibular advancement and setback, whereas the difference in pitch was statistically significant (*p* = 0.07, *p* = 0.8, *p* = 0.004, Mann–Whitney test).

## 4. Discussion

The results of the present study revealed no difference between the use of patient-specific CPDs and the manual positioning of the condyles in their respective fossa. Both methods resulted in the precise positioning of the ascending ramus. Several studies have discussed whether CPDs are beneficial or not. While some studies found no differences, others support the benefits of employing CPDs [11,21,22]. Renzi et al., for example, compared 15 patients treated with CPD and 15 conventionally operated patients. The condylar position in patients operated with CPDs differed from the preoperative position by less than two millimetres and two degrees, while differences in the control group were between two and four millimetres and four degrees [23]. However, CPDs are known to prolong and complicate the operation, which is why most surgeons favour the manual method [2,5].

For this reason, it is clearly desirable that we develop CPDs capable of reliable and stable surgical results without the disadvantages mentioned above. In a literature review by Chow et al., such CAD/CAM CPDs were revealed to be highly precise and eventually beneficial compared to manual positioning [5], which confirms our approach. The CPDs in the present study combine several positive properties.

First, the CPD is included in the final occlusal splint, which makes further steps unnecessary.

Second, the CPD is easy to handle, and the condyles may be guided into it precisely. This aspect was demonstrated by even greater reductions in operation duration in the intervention group (same distribution of bimaxillary operations, selective BSSOs, and displacements in both groups).

Finally, with these CPDs, it is possible not only to maintain the preoperative condylar position but to reproduce any pre-determined virtually planned one.

CAD/CAM devices were developed to transfer the virtually planned position of the displacement of the maxilla and the mandible to the operating room [18]. So far, however, only a few CAD/CAM devices allow for condylar positioning, and the described devices still focus on maintaining the preoperative condylar position. However, in some cases, the desired condylar position differs from this preoperative position [5,12]. With the CPDs designed in the present study, it was possible to adjust the ascending ramus and the condyle according to the virtually pre-planned position. The CPDs were printed in-house with a Formlabs three-dimensional printer. The printing costs seem to be low (one litre of surgical guide resin is about 300 euros and sufficient for multiple CPDs). However, determining the exact costs of in-house printing is difficult, as the printer is used for several devices (e.g., guided implantology, cutting guides for 3D-planned fibula transplants) [24].

While older studies concluded that CPDs are not superior to manual positioning, studies since 2003 focusing on postoperative temporomandibular joint function and relapses tend to find some benefit of employing CPDs [2,5,11]. Bettega et al. compared 10 patients in which the preoperative condylar position was secured by a 3D optical localizer with 10 patients conventionally operated (manual condylar positioning). The results of the study demonstrate significant differences between both groups in favour of optically guided condylar positioning. Patients suffered significantly less from TMD symptoms and less from skeletal relapses postoperatively [21]. Furthermore, other studies have demonstrated that the use of CPDs or CAD/CAM cutting and drilling guides could be beneficial in securing surgical results and thus reducing TMD symptoms [17,18,25,26]. This is partially supported by the results of our study, in which the mean deviation of the ascending ramus was slightly lower in the intervention compared to the control group. However, since the differences were not significant and TMD symptoms were not investigated, the present study failed to demonstrate any benefit of CPDs. Ha et al. described the role of the frontal–ramal inclination in facial symmetry. Especially in patients with facial asymmetries, it is important to bring the ascending rami to a laterally equal position during surgery. The results of the study demonstrated that virtual planning and CAD/CAM technologies were very useful in harmonizing the position of the ascending ramus [27]. CPDs as described in previous studies as well as in this study can be helpful to achieve this aim [5,28,29].

A limitation of the present study is the heterogeneous study collective, as we included patients with mandibular advancement and setback. Different skeletal classes were distributed equally to the study groups. Furthermore, we found no statistical difference in condylar translations between mandibular advancement and setback patients. Nevertheless, it cannot be ruled out that this may have influenced the study results or that there was a bias in patient recruitment. On the other hand, we did provide first evidence that the CPDs can be applied in both skeletal classes. Another study limitation is the small number of participants; a larger number of patient samples may reveal significant differences between CPDs and the manual positioning method. An evaluation of the long-term stability of the surgical result and the effect on TMD symptoms, clicking, and crepitation in the joint would have been an interesting aspect for further investigation. Another limitation is that the surgeries in the study were performed by different experienced maxillofacial surgeons. Different surgeons could have affected the accuracy of the condylar position, an aspect which was not investigated. However, this should have affected both groups equally. Furthermore, the temporomandibular joint has distinct structural complexities when compared to the rest of the mandibular bone, which has an impact on how it is represented on radiographic imaging [30]. Hence, the CBCT scan we used did not image the joint completely, which could have affected our results. Imaging in our study was the same for all patients, and this effect should be of only minor interest. Finally, different imaging methods (e.g., 2D X-ray scans, CBCT, and magnet resonance imaging) make it difficult to compare our results with other studies.

## 5. Conclusions

In summary, the present study revealed that postoperative results precisely matched the virtually planned positioning in both groups. The use of patient-specific CPDs resulted in no significant benefit compared with the manual repositioning of the condyles. However, it can be assumed that the described CPDs can be useful in difficult cases (Sunday bite) and for patients where the ascending ramus, respectively, the condyles need to be placed in a certain virtually planned position that differs from the preoperative situation. This could be very helpful in patients presenting facial asymmetries [27]. Furthermore, for trainees and less experienced surgeons, such CPDs may prove helpful towards achieving consistent and reliable results [31].

## Figures and Tables

**Table 1:** Patient characteristics.

	CPD (*n* = 14)	Control (*n* = 16)	*p*-Value
Mean age (in years) Min/max age	23 (SD ± 6.3) 18/41	26 (SD ± 7.2) 17/43	0.4
Gender (f/m)	9/5	9/7	0.7
Bimaxillary operation	12	13	0.3
Mandibular setback	8	7	
Mandibular advancement	4	6	
BSSO	2	3	
Mandibular setback	2	1	
Mandibular advancement	0	2	
Mean displacement of the mandible in mm (± SD)	3 (±3.2)	3.8 (±2.7)	0.2
Operation time (minutes)	181 (±54)	204 (±54)	0.2
Revision operation (*n*)	0	1	0.2

N: number of participants, CPD: condylar positioning device, m: male, f: female, SD: standard deviation, mm: millimetres, BSSO: bilateral sagittal split osteotomy.

**Table 2:** Differences with the ramus between the virtually planned position and postoperative result in millimetres.

	CPD/Control		
	Left/Right	Front/Back	Cranial/Caudal
Mean	0.8/1.1	0.8/1.3	1.3/1.6
SD	0.6/0.7	0.9/2.5	1.4/2.0
95%-CI			
CPD	0.5–1.0	0.5–1.2	0.7–1.8
Control	0.9–1.4	0.4–2.2	0.8–2.3
Difference of mean	0.3	0.5	0.3

* Significant CPD: condylar positioning device, SD: standard deviation, values are distances in mm.

**Table 3:** Differences in the ramus between the virtually planned position and postoperative result in degrees.

	CPD/Control		
	Pitch	Roll	Yaw
Mean	2.4/2.4	3.3/3.1	3.0/3.7
SD	1.6/2.0	2.9/2.6	1.9/3.2
95%-CI			
CPD	1.8–3.0	2.2–4.4	2.2–3.7
Control	1.7–3.1	2.2–4.1	2.6–4.8
Difference of mean	0	0.2	−0.7
*p*-Value	1.0	0.8	0.3

CPD: condyle positioning device, SD: standard deviation, values are degrees.

## Data Availability

The comprehensive clinical data and information are not publicly available because other, currently unpublished studies are based on them. However, these are available from the corresponding author upon reasonable request.

## References

[1] Lee Y.C., Sohn H.B., Park Y.W., Oh J.-H. (2022). Evaluation of postoperative changes in condylar positions after orthognathic surgery using balanced orthognathic surgery system. Maxillofac. Plast. Reconstr. Surg..

[2] Costa F., Robiony M., Toro C., Sembronio S., Polini F., Politi M. (2008). Condylar positioning devices for orthognathic surgery: Please includeA literature review. Oral. Surg. Oral. Med. Oral. Pathol. Oral. Radiol. Endod..

[3] Ueki K., Nakagawa K., Marukawa K., Takazakura D., Shimada M., Takatsuka S., Yamamoto E. (2005). Changes in condylar long axis and skeletal stability after bilateral sagittal split ramus osteotomy with poly-L-lactic acid or titanium plate fixation. Int. J. Oral. Maxillofac. Surg..

[4] Kaur A., Rattan V., Rai S., Singh S.P., Kalra P., Sharma S. (2022). Changes in condylar position after orthognathic surgery and its correlation with temporomandibular symptoms (TMD)—A prospective study. J. Craniomaxillofac. Surg..

[5] Chow W., He Z., Liu Y., Song J., Xu C., Luo E. (2022). Intraoperative condylar positioning techniques on mandible in orthognathic surgery. Orthod. Craniofac Res..

[6] Mazzone N., Matteini C., Incisivo V., Evaristo B. (2009). Temporomandibular joint disorders and maxillomandibular malformations: Role of condylar “repositionin” plate. J. Craniofac Surg..

[7] Sanroman J.F., Gonzalez J.M.G., del Hoyo J.A. (1998). Relationship between condylar position, dentofacial deformity and temporomandibular joint dysfunction: An MRI and CT prospective study. J. Cranio-Maxillofac. Surg..

[8] Stavropoulos F., Dolwick M.F. (2003). Simultaneous temporomandibular joint and orthognathic surgery: The case against. J. Oral. Maxillofac. Surg..

[9] Landes C.A., Sterz M. (2003). Proximal segment positioning in bilateral sagittal split osteotomy: Intraoperative controlled positioning by a positioning splint. J. Oral. Maxillofac. Surg..

[10] Kim M.I., Kim J.H., Jung S., Park H.-J., Oh H.-K., Ryu S.-Y., Kook M.-S. (2015). Condylar positioning changes following unilateral sagittal split ramus osteotomy in patients with mandibular prognathism. Maxillofac. Plast. Reconstr. Surg..

[11] Ellis E. (1994). Condylar Positioning Devices for Orthognathic Surgery—Are They Necessary?. J. Oral. Maxillofac. Surg..

[12] Ueki K., Moroi A., Sotobori M., Ishihara Y., Marukawa K., Takatsuka S., Yoshizawa K., Kato K., Kawashiri S. (2012). A hypothesis on the desired postoperative position of the condyle in orthognathic surgery: A review. Oral. Surg. Oral. Med. Oral. Pathol. Oral. Radiol..

[13] Helm G., Stepke M.T. (1997). Maintenance of the preoperative condyle position in orthognathic surgery. J. Craniomaxillofac. Surg..

[14] Cortese A., Chandran R., Borri A., Cataldo E. (2019). A Modified Novel Technique for Condylar Positioning in Mandibular Bilateral Sagittal Split Osteotomy Using Computer-Assisted Designed and Computer-Assisted Manufactured Surgical Guides. J. Oral. Maxillofac. Surg..

[15] Abdel-Moniem Barakat A., Abou-ElFetouh A., Hakam M.M., El-Hawary H., Abdel-Ghany K.M. (2014). Clinical and radiographic evaluation of a computer-generated guiding device in bilateral sagittal split osteotomies. J. Craniomaxillofac. Surg..

[16] Abou-ElFetouh A., Barakat A., Abdel-Ghany K. (2011). Computer-guided rapid-prototyped templates for segmental mandibular osteotomies: A preliminary report. Int. J. Med. Robot..

[17] Polley J.W., Figueroa A.A. (2013). Orthognathic positioning system: Intraoperative system to transfer virtual surgical plan to operating field during orthognathic surgery. J. Oral. Maxillofac. Surg..

[18] Zinser M.J., Mischkowski R.A., Sailer H.F., Zöller J.E. (2012). Computer-assisted orthognathic surgery: Feasibility study using multiple CAD/CAM surgical splints. Oral. Surg. Oral. Med. Oral. Pathol. Oral. Radiol..

[19] Lee C.H., Cho S.W., Kim J.W., Ahn H.J., Kim Y.H., Yang B.E. (2019). Three-dimensional assessment of condylar position following orthognathic surgery using the centric relation bite and the ramal reference line: A retrospective clinical study. Medicine.

[20] Bockmann R., Meyns J., Dik E., Kessler P. (2014). The modifications of the sagittal ramus split osteotomy: A literature review. Plast. Reconstr. Surg. Glob. Open.

[21] Bettega G., Cinquin P., Lebeau J., Raphaël B. (2002). Computer-assisted orthognathic surgery: Clinical evaluation of a mandibular condyle repositioning system. J. Oral. Maxillofac. Surg..

[22] Bettega G., Dessenne V., Raphael B., Cinquin P. (1996). Computer-assisted mandibular condyle positioning in orthognathic surgery. J. Oral. Maxillofac. Surg..

[23] Renzi G., Becelli R., Di Paolo C., Iannetti G. (2003). Indications to the use of condylar repositioning devices in the surgical treatment of dental-skeletal class III. J. Oral. Maxillofac. Surg..

[24] Vollmer A., Saravi B., Breitenbuecher N., Mueller-Richter U., Straub A., Šimić L., Kübler A., Vollmer M., Gubik S., Volland J. (2023). Realizing in-house algorithm-driven free fibula flap set up within 24 hours: A pilot study evaluating accuracy with open-source tools. Front. Surg..

[25] Li K., Li J., Du W., Xu C., Ye B., Luo E. (2020). Computer-Aided Design and Manufacturing Cutting and Drilling Guides with Prebent Titanium Plates Improve Surgical Accuracy of Skeletal Class III Malocclusion. Plast. Reconstr. Surg..

[26] Landes C.A. (2004). Proximal segment positioning in bilateral sagittal split osteotomy: Intraoperative dynamic positioning and monitoring by sonography. J. Oral. Maxillofac. Surg..

[27] Ha S.H., Meny A.H., Jeong C.G., Yeo J.H., Baek S.H., Choi J.Y. (2023). The Accuracy and Stability of Intentional Change of Frontal-Ramal Inclination in Orthognathic Surgery for Facial Asymmetry Patients. J. Craniofac Surg..

[28] Kim J.W., Kim J.C., Cheon K.J., Cho S.W., Kim Y.H., Yang B.E. (2018). Computer-Aided Surgical Simulation for Yaw Control of the Mandibular Condyle and Its Actual Application to Orthognathic Surgery: A One-Year Follow-Up Study. Int. J. Environ. Res. Public Health.

[29] Park J.H., Lee Y.B., Kim S.Y., Kim H.J., Jung Y.-S., Jung H.-D. (2019). Accuracy of modified CAD/CAM generated wafer for orthognathic surgery. PLoS ONE.

[30] Tecco S., Baldini A., Nakas E., Primozic J. (2017). Interceptive Orthodontics and Temporomandibular Joint Adaptations: Such Evidences?. Biomed. Res. Int..

[31] Savoldelli C., Chamorey E., Bettega G. (2018). Computer-assisted teaching of bilateral sagittal split osteotomy: Learning curve for condylar positioning. PLoS ONE.

